# Artificial Infestation of *Sarcoptes scabiei* (Acari: Sarcoptidae) in Rabbits Exhibits Progressive Pathological Changes, Apoptosis, and Keratinization in the Skin

**DOI:** 10.3390/ijms24032187

**Published:** 2023-01-22

**Authors:** Ke Guan, Jing Xu, Xiaobin Gu, Ran He, Yue Xie, Bo Jing, Xuerong Peng, Guangyou Yang

**Affiliations:** 1 Department of Parasitology, College of Veterinary Medicine, Sichuan Agricultural University, Chengdu 611130, China; 2 Department of Chemistry, College of Life and Basic Science, Sichuan Agricultural University, Chengdu 611130, China

**Keywords:** *Sarcoptes scabiei*, pathological changes, rabbits, skin, apoptosis, keratinization

## Abstract

*Sarcoptes scabiei* (*S. scabiei*) is an ectoparasite that can infest humans and 150 mammalian host species, primarily causing pruritus, crust, and alopecia. However, neither the pathological process of host skin under *S. scabiei* infection nor the mechanism of *S. scabiei* infection in regulating apoptosis and keratinization of host skin has been studied yet. In this study, a total of 56 rabbits were artificially infested with *S. scabiei*, and the skin samples were collected at seven different time points, including 6 h, 12 h, day 1, day 3, 1 week, 4 weeks, and 8 weeks, whereas a group of eight rabbits served as controls. We measured epidermal thickness by H&E staining, observed the skin ultrastructure by electron microscopy, and detected the degree of skin apoptosis by TUNEL staining. The level of transcription of genes related to apoptosis and keratinization was detected by quantitative real-time PCR (qRT-PCR), and the level of Bcl-2 protein expression was further detected using immunohistochemistry. Our results showed that, with increased infestation time, the epidermal layer of the rabbit skin exhibited significant thickening and keratinization, swollen mitochondria in the epidermal cells, and increased skin apoptosis. The level of caspase-1, 3, 8, 10, 14, and Bcl-2 mRNA expression was increased, whereas the level of keratin 1 and 5 was decreased after *S. scabiei* infestation. In conclusion, *S. scabiei* infestation causes thickening of the epidermis, which may be related to apoptosis-induced proliferation and skin keratinization.

## 1. Introduction

*Sarcoptes scabiei* (De Geer) (*S. scabiei*) is an ectoparasite distributed worldwide and infests humans and over 148 domestic and wild mammalian hosts belonging to 39 families and 12 orders [1]. In addition, *S. scabiei* causes skin diseases characterized by pruritus, erythema, crusty dermatitis, and alopecia [1]. In clinical practice, *S. scabiei* infestation is prone to being misdiagnosed due to similar signs shared with other dermatology diseases, including allergic dermatitis and fungal skin infestations [2]. The predecessors’ research investigating pathological changes in *S. scabiei* infestation has primarily focused on the observation of clinical cases in humans [3] and domestic animals (e.g., rabbits, pigs, and sheep) [4,5,6,7,8,9], and wild animals (e.g., deer, koalas, red foxes, raccoons, wolves, bears, and wombats) [10,11,12,13,14]. However, studies on the dynamic pathological changes in *S. scabiei*-infested animals remain limited. At present, the artificial infestation of rabbits with *S. scabiei* isolated from dogs and wild rabbits [15,16,17], the artificial infestation of goats with *S. scabiei* isolated from wild ibex [18], and the artificial infestation of pigs with *S. scabiei* var. *suis* [19] have been studied.

Previous studies have demonstrated that *S. scabiei* infestation in dogs and pigs can cause peripheral blood leukocyte apoptosis [7,20]. Caspases, a family of intracellular cysteine proteases, can be triggered to cause apoptosis [21]. Bcl-2 is a classic antiapoptotic protein [22]. As the primary immune barrier, the skin can generate several antimicrobial proteins from keratinocyte differentiation, which mainly attack bacteria, viruses, fungi, and parasites [23,24]. The epidermal barrier is constructed by corneocytes, formed through programed cell death termed cornification [25]. In host skin wound healing, keratinization plays a crucial role in re-epithelization [26]. However, neither the pathological process that occurs in the host skin under *S. scabiei* infestation nor the mechanism by which an *S. scabiei* infestation regulates apoptosis and keratinization of the host skin have been studied to date. In this study, we artificially infested rabbits with *S. scabiei* and studied the progressive clinical characteristics following *S. scabiei* infestation, the pathological changes in the skin, and the potential mechanisms of skin apoptosis and keratinization.

## 2. Results

### 2.1. Clinical Signs of Rabbits Infested with S. scabiei

**Figure 1:**
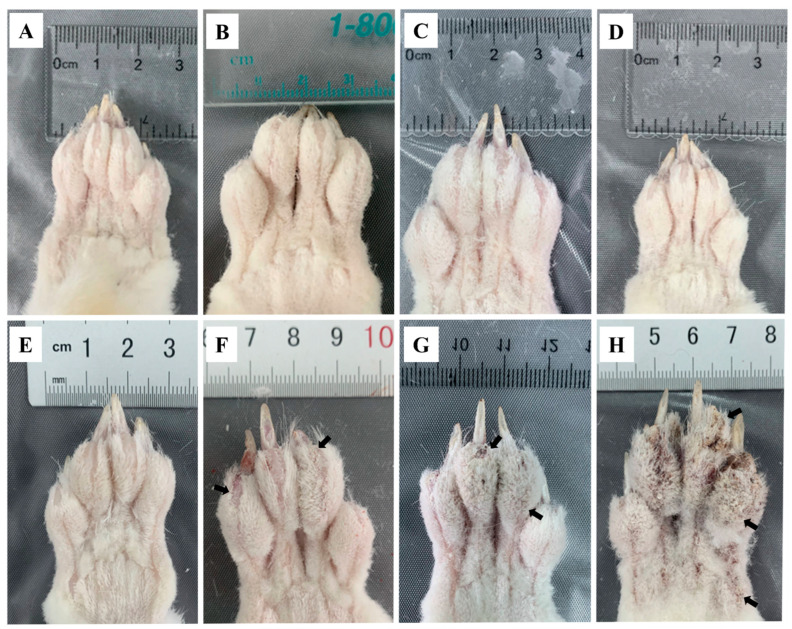
Clinical changes in rabbits infested with *S. scabiei* (**A**) control, (**B**) 6 hpi, (**C**) 12 hpi, (**D**) 1 dpi, (**E**) 3 dpi, (**F**) 1 wpi, (**G**) 4 wpi, (**H**) 8 wpi. Arrows point to the crust.

The toes of the infested rabbits displayed swelling at 6 hpi (Figure 1B) and faded away by 12 hpi (Figure 1C) compared to the control group (Figure 1A). From 1 dpi to 3 dpi, no abnormal ocular changes were observed on the toes (Figure 1D,E). At 1 wpi, some crust appeared on the end of the toes (Figure 1F, arrows). The crust became thicker and spread to the entire toes after 4 wpi (Figure 1G, arrows). At 8 wpi, the toes were covered with a thick layer of crust (Figure 1H, arrows), and the obvious crust was also observed on the rabbits’ noses, mouths, and ear edges. After the artificial infestation, the rabbits presented with itching activities, such as shaking legs and biting feet. These activities became more intense with an increase in infestation time.

### 2.2. Progressive Pathological Changes in the Skin after Infestation

**Figure 2:**
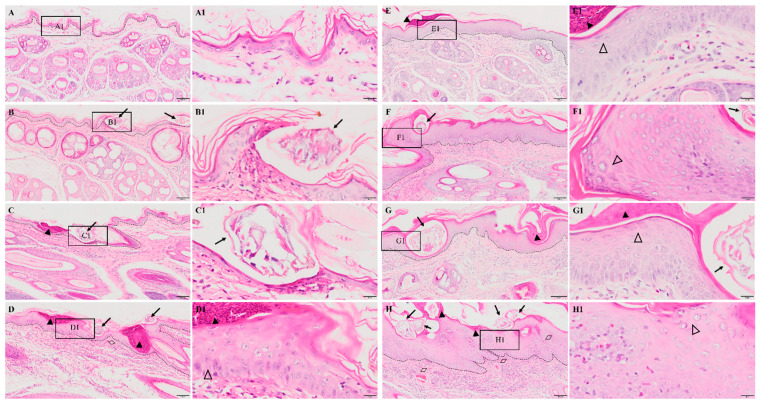
Pathological changes in the posterior toe skin in rabbits. (**A**) control, (**B**) 6 hpi, (**C**) 12 hpi, (**D**) 1 dpi, (**E**) 3 dpi, (**F**) 1 wpi, (**G**) 4 wpi, (**H**) 8 wpi. The black dotted line in figures (**A**–**H**) indicates the epidermis–dermis junction. Figures (**A1**–**H1**) (scale bar = 20 μm) represent the enlarged figure of the black frame in figures (**A**–**H**) (scale bar = 100 μm). The arrows point to the *S. scabiei*, the solid triangles point to the scab, the hollow triangles point to hyperkeratosis, and the rhombuses point to congestion and hemorrhaging.

**Figure 3:**
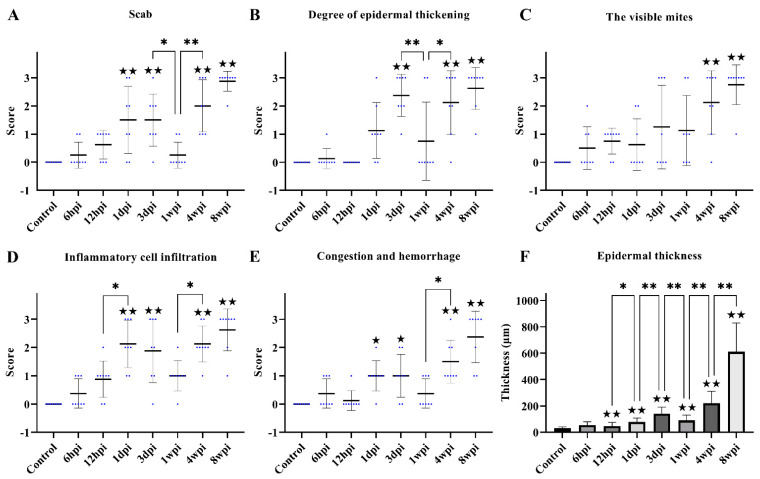
Scoring of pathological changes. (**A**) Scab, (**B**) degree of epidermal thickening, (**C**) the visible mites, (**D**) inflammatory cell infiltration, (**E**) congestion and hemorrhage, and (**F**) epidermal thickness. (**A**–**E**) shows the score of skin pathological changes according to the Table 1, blue dots represent the number of rabbits, and the black line represent the average score. (**F**) shows epidermal thickness measured by Image-Pro Plus 5.1 software, randomly measuring at least 30 widths of each H&E-stained skin section along the epidermis. Data were represented as the mean ± standard deviation. (M ± SD). The horizontal line indicates a significant adjacent pairwise group difference (* *p* < 0.05 and ** *p* < 0.01). ★ on the bar represents a significant difference between the specific experimental group and the control group (★ *p* < 0.05 and ★★ *p* < 0.01).

No obvious pathological damage was observed in the control group (Figure 2A). At 6 hpi, *S. scabiei* invaded the stratum corneum and stratum lucidum (Figure 2B, arrows). The cuticle was destroyed and a mass of inflammatory exudate was observed in the dermis (Figure 2B). The dermis near the *S. scabiei* was infiltrated with neutrophils and lymphocytes (Figure 2B,C). At 12 hpi, obvious scabs had formed in the epidermis (Figure 2C, solid triangles) and there was an increased number of inflammatory cells in the dermis (primarily neutrophils and plasma cells) (Figure 2C). At 1 dpi, epidermal thickening and hyperkeratosis were visible in the epidermis (Figure 2D and Figure 3B, hollow triangles).

After 3 dpi, similar pathological characteristics were observed, including epidermal damage, extensive inflammatory exudate, scab formation, thickening, and hyperkeratosis of the epidermis (Figure 2E–H and Figure 3A–E). The inflammatory cells (mainly neutrophils and plasma cells) and the congestion and hemorrhage spots increased along with the progression of the infestation (Figure 2E–H, rhombuses, and Figure 3E). At 8 wpi, the epidermis was the thickest (Figure 3F). In total, the scores of all pathological indexes increased from 6 hpi to 3 dpi, and then began to increase again after 1 wpi of decline (Figure 3A–E). The scab and degree of the epidermal thickening scores decreased from 3 dpi to 1 wpi and increased from 1 wpi to 4 wpi (Figure 3A,B). The scores of inflammatory cell infiltration increased from 12 hpi to 1 dpi and from 1 wpi to 4 wpi (Figure 3D). The scores for congestion and hemorrhage increased from 1 wpi to 4 wpi (Figure 3E). The epidermal thickness increased from 12 hpi to 1 dpi and continued to increase significantly in pairwise comparisons (Figure 3F).

### 2.3. Observations of Ultrastructural Pathological Changes

**Figure 4:**
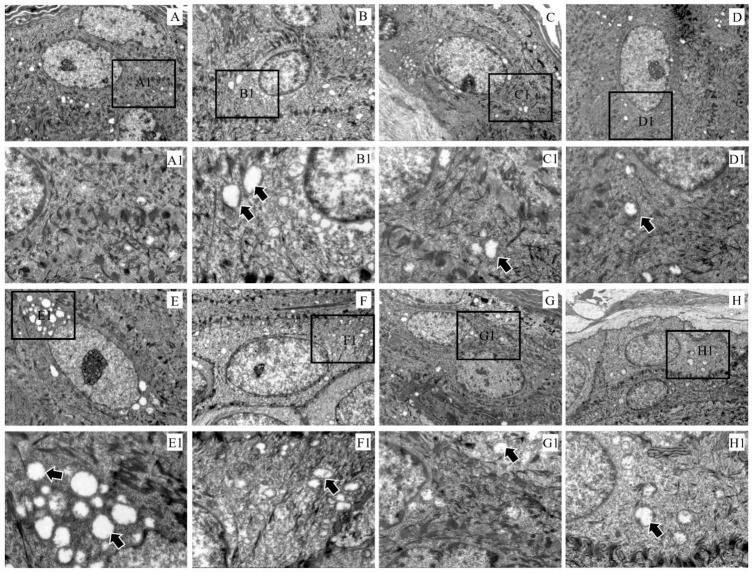
Ultramicropathology of the epidermis of rabbit skin. (**A**) Control, (**B**) 6 hpi, (**C**) 12 hpi, (**D**) 1 dpi, (**E**) 3 dpi, (**F**) 1 wpi, (**G**) 4 wpi, (**H**) 8 wpi. Figures (**A1**–**H1**) (×3000) represent the enlarged figure of the black frame in figures (**A**–**H**) (×8000). The arrows point to mitochondrial swelling.

Compared with the control group, the electron density in the keratinocyte cytoplasm became sparse and the mitochondria were swollen (Figure 4A–H). In particular, the mitochondrial vacuolation in the epidermal layer was the most severe at 3 dpi (Figure 4E).

### 2.4. Detection of Skin Apoptosis by TUNEL Staining

**Figure 5:**
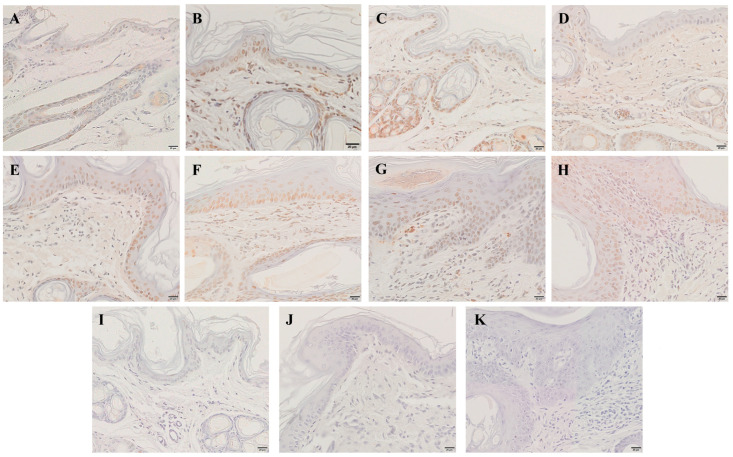
TUNEL staining for the apoptosis of skin cells. (**A**) TUNEL staining of the skin before artificial infestation (control). (**B**–**H**) TUNEL staining for skin artificially infested for 6 h, 12 h, 1 d, 3 d, 1 w, 4 w, and 8 w. (**I**–**K**) Negative control. Brownish-yellow nuclei cells are the TUNEL-positive cells. Scale bar = 20 μm.

Compared with the control group, the apoptotic cells began to appear in the epidermis at 6 hpi (Figure 5B), and the number of apoptotic cells gradually increased with infestation time (Figure 5B–H). Apoptotic cells were also identified in the dermis at 12 hpi (Figure 5C), and the number increased with infestation time (Figure 5C–H). 

### 2.5. Transcription of Genes Related to Skin Apoptosis and Keratinization 

**Figure 6:**
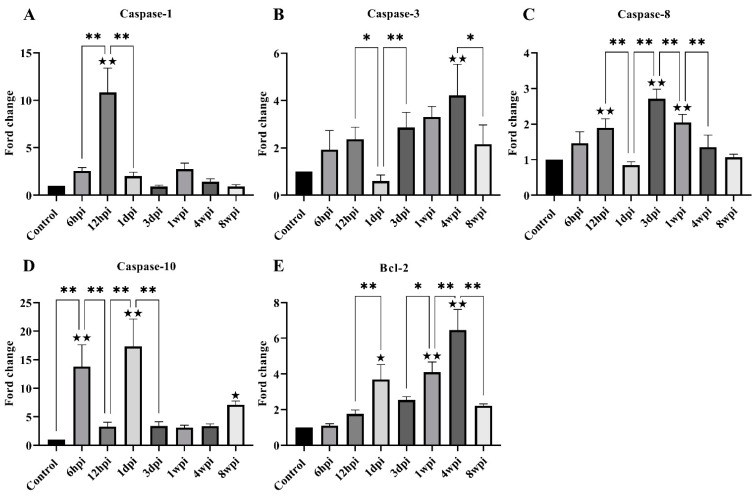
The level of mRNA expression of genes related to skin apoptosis. (**A**) Caspase-1, (**B**) Caspase-3, (**C**) Caspase-8, (**D**) Caspase-10, (**E**) Bcl-2. Data are represented as the mean ± standard deviation (M ± SD). A one-way ANOVA analysis was used to analyze the variances between the groups. The horizontal line indicates a significant adjacent pairwise group difference (* *p* < 0.05 and ** *p* < 0.01). ★ on the bar represents a significant difference between the specific experimental group and the control group (★ *p* < 0.05 and ★★ *p* < 0.01).

For genes associated with apoptosis, the level of caspase-1, caspase-3, caspase-8, caspase-10, and Bcl-2 expression was investigated. The level of caspase-1 mRNA expression was found to be significantly increased at 12 hpi, and then significantly decreased at 1 dpi (Figure 6A). The overall caspase-3 and caspase-8 mRNA levels increased parabolically along with the infestation time but significantly decreased at 1 dpi and 8 wpi (Figure 6B,C). The level of caspase-10 mRNA was significantly increased at 6 hpi, 1 dpi, and 8 wpi compared with the control group (Figure 6D). The level of Bcl-2 mRNA expression was significantly increased at 1 dpi, 1 wpi, and 4 wpi compared with the control group (Figure 6E).

**Figure 7:**
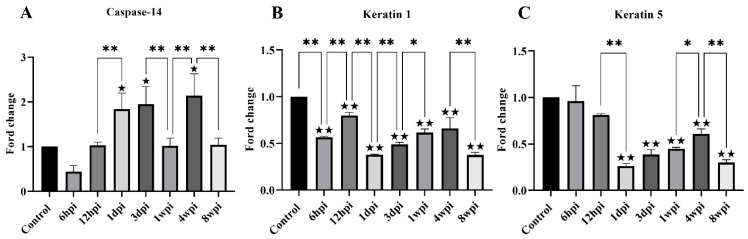
The level of mRNA expression of genes related to skin keratinization. (**A**) Caspase-14, (**B**) Keratin 1, (**C**) Keratin 5. Data are represented as the mean ± standard deviation (M ± SD). A one-way ANOVA analysis was used to analyze the variances between the groups. The horizontal line indicates a significant adjacent pairwise group difference (* *p* < 0.05 and ** *p* < 0.01). ★ on the bar represents a significant difference between the specific experimental group and the control group (★ *p* < 0.05 and ★★ *p* < 0.01).

For genes associated with keratinization, the level of keratin-1, keratin-5, and caspase-14 mRNA expression was studied. The level of caspase-14 mRNA was significantly increased at 1 dpi, 3 dpi, and 4 wpi compared with the control group (Figure 7A). Overall, the level of keratin-1 mRNA expression was significantly decreased compared with the control group, with slight fluctuations between the experimental groups (Figure 7B). The level of keratin-5 mRNA expression began to decrease due to artificial infestation and was significantly decreased at 1 dpi compared with the control group (Figure 7C).

### 2.6. Immunohistochemistry

**Figure 8:**
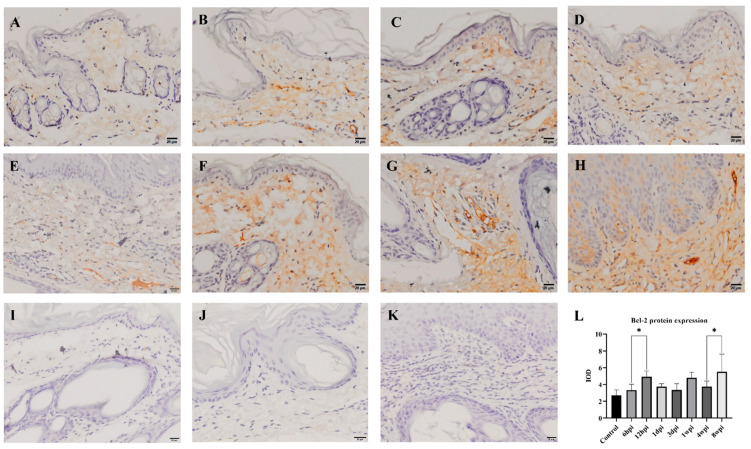
Bcl-2 protein expression was detected by immunohistochemical staining. (**A**) Control, (**B**) 6 hpi, (**C**) 12 hpi, (**D**) 1 dpi, (**E**) 3 dpi, (**F**) 1 wpi, (**G**) 4 wpi, (**H**) 8 wpi, (**I**–**K**) negative control, (**L**) integral optical density (IOD) of Bcl-2 protein expression. Brownish-yellow staining indicates Bcl-2 antigenicity (cytoplasmic staining). The IOD values of Bcl-2 protein expression are represented as the mean ± standard deviation (M ± SD). A one-way ANOVA was used to analyze the variances between groups. The horizontal line indicates a significant adjacent pairwise group difference (* *p* < 0.05). Scale bar = 20 μm.

The level of antiapoptotic Bcl-2 protein expression in the skin was detected by immunohistochemistry, and the cytoplasm of positive cells was dyed brownish yellow. During 6 hpi to 4 wpi, the Bcl-2 protein was predominantly expressed in the dermis (Figure 8B–G). At 8 weeks, Bcl-2 proteins were expressed in the epidermis and dermis (Figure 8H). According to the average IOD values, the level of Bcl-2 protein expression was significantly increased at 12 hpi and 8 wpi when compared to the control group (*p* < 0.01) (Figure 8L).

## 3. Discussion

The main manifestations of *S. scabiei* infestation include pruritus, erythema, hyperkeratosis, seborrhea, and alopecia [12]. However, the clinical signs of humans and animals with *S. scabiei* infestation are not obvious during the initial stage. The onset time of the visible clinical signs and the clinical characteristics also differ between hosts [16,27]. The delayed appearance of clinical signs in the host (most likely caused by immune evasion or suppression) has hindered treatment efforts. Previous studies have shown that, for artificially infested goats (*Capra pyrenaica*), the crusts appeared on the heads at 46 dpi [18]. In contrast, the crusts were not visible for rabbits and pigs until 2 wpi [15] and 6 wpi [28], respectively. For humans, the incubation period for the initial infestation ranges from 3 to 6 weeks, and it takes approximately 4 weeks or longer for clinical signs to become apparent [29]. Our study investigated the changes in the clinical signs of rabbits artificially infested with *S. scabiei* for 8 weeks. The visible clinical signs appeared at 1 wpi, earlier than the rabbits infested with *S. scabiei* (3600/per rabbit) isolated from wild rabbits [15]. Although *S. scabiei* has adapted to the obligated parasitic life in the epidermal skin in morphology, detoxification, and nutrition metabolisms [30], it is believed that *S. scabiei* from different hosts have a “high degree of host specificity and low degree of cross-infectivity” [31]. Therefore, the early appearance of visible clinical signs in rabbits may be related to the long-term adaption of *S. scabiei* in their native rabbit hosts.

It has previously been reported that subspecies of *S*. *scabiei* in different hosts have different preferences for invasion sites. In our study, the appearance of early crusts began to appear on the tiptoe of the rabbits at 1 wpi and was subsequently gradually observed on the noses, mouths, and ear edges of the rabbits after 4 wpi. We speculate that rabbits prefer to scratch their nose and ears using the legs and bite the limbs when they feel itching, leading to the crusts observed on these sites. Clinically, obvious signs (the appearance of crusts) were originally observed on the limbs in naturally infested rabbits, which is why we simulate natural infestation through artificially infested rabbit limbs. *S*. *scabiei* prefers to invade the hands, wrists, feet, ankles, palms, flexor surfaces of the knees, elbows, neck, and genital areas of humans [32,33], the head of goats [18], and the hind limbs, sciatic area, and ear base of canids [34,35,36]. To date, the reason that *S. scabiei* prefers to infect these regions remains unknown and should be further studied. We speculate that the different invasion preferences of *S. scabiei* may be related to the differences in skin structure, lipid content, and other characteristics of different hosts. Rabbits infested with *S. scabiei* exhibit signs similar to those of crusted scabies in humans [37] without other treatment, where the porcine model requires treatment with the glucocorticoid dexamethasone [19,38]. Moreover, rabbits are the natural host of *S. scabiei*, relatively cheap compared with goat, pig, or dog models, and, most importantly, obvious crusts appear as early as 1 wpi. In addition, rabbit and human skin respond similarly to aging, delayed healing, and various topical drugs [39], making rabbits an optimal animal model for studying the mechanisms associated with the development of crusted scabies and the treatment of this disease.

*S. scabiei* secretes and excretes proteins to digest and break down the cuticle of the host skin, allowing mites to reach the epidermis [40] and establishing the complete life cycle [2]. Our study found that *S. scabiei* invaded the epidermis at 6 hpi and massive inflammatory exudate was observed in regions adjacent to *S. scabiei* in the epidermis. At 3 dpi, the level of inflammatory cell infiltration was decreased and the epidermal layer displayed significant thickening. The decreased inflammatory cell infiltration at 3 dpi may also contribute to *S. scabiei* survival by regulating host skin cells’ innate and adaptive immune systems [41]. As the cuticle of the skin is destroyed by the invasion of *S. scabiei*, the thickened keratinized layers at 1 dpi may represent a compensatory mechanism by which the tissue responds to the defective epidermal barrier [25]. 

Mitochondria are important regulators of skin physiology, and mitochondrial metabolism regulates keratocyte differentiation by generating reactive oxygen species (ROS) within the mitochondria [42]. In our study, *S. scabiei* infestation caused swelling of mitochondria in skin cells. Mitochondrial damage was the most extensive at 6 hpi and 3 dpi, and severe vacuolation occurred. We speculated that, as the skin barrier becomes damaged by the invasion of *S. scabiei*, the skin undergoes repair, which requires energy and causes mitochondrial depletion and abnormalities. When mitochondria swell abnormally, cytochrome C (Cyt-c) is released, resulting in caspase-mediated cell death [43], further accelerating the host skin’s excessive keratinization.

It has been widely accepted that apoptosis is a distinct and important mode of “programmed” cell death [44]. During apoptosis, cells display several morphological and biochemical characteristics, including chromatin condensation, shrinkage of the cell and nuclear membrane, and fragmentation of the cell membrane [45]. A TUNEL assay is a widely used method to detect apoptotic cells that undergo extensive DNA degradation [45]. Previous studies have shown that *S. scabiei* can invade the skin in less than an hour [46]. Female mites begin to penetrate the skin within about 20 min and semi-dive or fully dive into the newly formed burrow of skin within 1 h [46]. Males, nymphs, and larvae begin burrowing less than five minutes after being placed on the skin [46]. In our study, the TUNEL staining was increased following the artificial infestation of *S. scabiei*, and we speculate that the invasion of *S. scabiei* may destroy the cell of the host epidermis and lead to the apoptosis of the host skin.

Apoptosis is a co-ordinated and energy-dependent process involving activating a group of cysteine proteases termed “caspases”, which can activate the inflammasome and induce programed cell death [47,48]. Moreover, caspases can trigger apoptosis-induced proliferation (AIP), which plays an important role in wound healing and tissue regeneration [49]. Bcl-2 and caspases represent the key genes for regulating apoptosis [50]. The Bcl-2 protein has the function of inhibiting cytochrome C release, which functions as an antiapoptotic protein [51]. A significant increase in the apoptosis of leukocytes was observed in the peripheral blood of dogs and pigs infested with *S. scabiei* [7,20]. However, there remains a lack of research regarding the level of apoptosis in the skin, which is the direct invasive site of *S. scabiei*.

To elucidate the mechanism of skin apoptosis during *S. scabiei* infestation, we detected the caspase-1, caspase-3, caspase-8, caspase-10, and Bcl-2 transcription levels. Caspase-1 is an inflammatory caspase that can initiate apoptosis [52] and, in caspase-1 knockout mice, apoptosis was no more pronounced than inflammatory and related processes [53]. In this study, caspase-1 expression significantly increased at 12 hpi, accompanied with the observation of *S. scabiei* in the epidermis at 6 hpi. We postulated that the observations of inflammation following *S. scabiei* infestation may coincide with increased expression of caspase-1, which may initiate skin apoptosis. Moreover, the expression of the overall levels of caspase-3, caspase-8, and caspase-10 mRNA were increased with the infestation time. Caspase-8 and caspase-10 are initiators of extrinsic (death ligand) apoptotic pathways or intrinsic (mitochondrial) apoptotic pathways [54,55], while caspase-3 is activated by either caspase-8 or caspase-10 and served as the key executioner caspase apoptotic pathway [56,57]. According to the pathological changes in the skin, the epidermal layer displayed continuous thickening. The expression of caspase-8 and caspase-10 started to increase since 6hpi, and caspase-3 kept a steady increase along with the infestation time, which suggests that the invasion of *S. scabiei* may induce the increase in caspase-8 and caspase-10, and initiate the skin apoptosis by increase in caspase-3. One previous study has demonstrated that the parasite enhanced Bcl-2 to counteract the host’s apoptosis defense [58]. Our study further showed that the levels of Bcl-2 mRNA and protein expression were increased after *S. scabiei* infestation, suggesting that the host’s Bcl-2 protein can inhibit skin apoptosis, which benefits wound healing of the skin.

When *S. scabiei* invades the host, it escapes the host’s immune defenses, leading to the host skin’s excessive proliferation or apoptosis [40]. During homeostasis, the outer skin barrier’s cornified layers are continuously shed by desquamation, a unique programed cell death called keratinization [25]. When the skin has excessive desquamation, it may lead to the formation of crust. Caspase-14 is a nonapoptotic caspase involved in keratinocyte differentiation and cornification of the skin, which is a predominant caspase in the epidermal stratum corneum [59]. Caspases-14-deficient mice develop parakeratosis (persistent nuclei in keratinized regions) when epidermal hyperplasia is present, and abnormal keratinization occurs following acute barrier disruption [60]. Our findings revealed that caspase-14 expression was significantly increased at 1 dpi, 3 dpi, and 4 wpi, indicating that caspase-14 may be involved in skin epidermal differentiation caused by *S. scabiei* invasion. During the keratinocyte differentiation process, the spinous layer cells can no longer divide and express typical differentiation markers (e.g., keratin 1 and caspase-14), and the basal layer expresses Keratin 5 [25]. Keratin 1 is critical for maintaining skin integrity, and a Keratin 1 knockdown leads to cell proliferation, migration, and contraction to synthetic transformation [61]. In our study, the Keratin 1 and 5 mRNA expression level was significantly decreased. We postulated that the invasion of *S. scabiei* may cause a significant increase in skin apoptosis, thereby leading to keratinization. 

## 4. Materials and Methods

### 4.1. Mites and Animals

The *S. scabiei* var. *cuniculi* used in this study was derived from a clinically affected New Zealand White rabbit and was then maintained in New Zealand White rabbits. Ten seeder rabbits with severe mange lesions were euthanized and served as sources of mites. The hair on lesioned limbs was shaved, and the remainder hair was burned with great care under an alcohol burner. The processed limbs were placed in 10 cm Petri dishes at 38 °C to encourage mites to migrate out of the limbs. The mites, without considering their sexes and life stages, were collected every 1 h and maintained as 0.0017~0.0020 g per sample (approximately 2000 mixed life-cycle stage live mites) [62]. A total of 64 healthy 3-month-old New Zealand White rabbits (weight 2.24 g ± 0.15 kg) were purchased from Chengdu Dossy Biotechnology Co., Ltd. (with no history of mange). Animals were housed individually and kept under observation during an acclimatization period of 1 week before the experiment.

### 4.2. Experimental Design and Collection of Skin Samples

A total of 64 rabbits were randomly divided into 8 groups of eight rabbits. Seven groups of rabbits were artificially infested, whereas one group of rabbits was not infested and euthanized as a control group. To perform the artificial infestation, we shaved the hair on the toes of the hind feet to expose the skin on the toes. Approximately 2000 *S. scabiei* were gently placed on the bare skin of the toes and wrapped with gauze to prevent the mites from falling off, and the gauze was removed after 1 h; the toes of the rabbits in the control group were dealt with in the same way but without mite exposure. For each of the seven infestation groups, at 6 h (6 hpi), 12 h (12 hpi), 1 day (1 dpi), 3 days (3 dpi), 1 week (1 wpi), 4 weeks (4 wpi), and 8 weeks (8 wpi), one group of rabbits was euthanized. The toe skin was peeled off using small scissors. The skin samples were divided into three parts: one part of the skin was immediately put into liquid nitrogen, one part was fixed in a 4% paraformaldehyde solution, and the other part was fixed in the dark in an electron microscopy fixative solution at room temperature for 2 h, and then cut into pieces (about 1 mm^3^) with a scalpel, placed into a new electron microscopy fixative solution, and stored at 4 °C.

### 4.3. Observation of Pathological Changes and Measurement of the Thickness of the Epidermal Layer

To observe the dynamic pathological changes in rabbits infested by *S. scabiei*, the skin samples fixed in a 4% paraformaldehyde solution were further processed and embedded in paraffin, sectioned into 5 mm thick sections, and stained with hematoxylin and eosin (H&E) [63]. The skin tissue sections were observed and photographed using the slide scanner Olympus VS120 (Olympus, Japan).

The skin tissue pathological damage was scored using the proposed scoring system [64,65] and modified as shown in Table 1. Each H&E-stained skin section was measured randomly at least 30 widths along the epidermis to determine epidermal thickness using Image-Pro Plus 5.1 software (Media Cybernetics, Bethesda, MD, USA).

### 4.4. Observation of Ultrastructural Pathology

The skin samples preserved in the electron microscopy fixative solution were taken out and rinsed in 4% glutaraldehyde in Sorensen’s phosphate buffer (SPB) (pH 7.4). Next, samples were post-fixed with 1% osmium tetroxide for 2 h, dehydrated in graded ethanol (30%, 50%, 70%, 80%, 95%, and 100%), and embedded in EMBed 812. The samples were cut into 60–80 nm thin sections on the ultramicrotome. The thin sections were mounted on 150 meshes cuprum grids with formvar film, stained with 2% uranium acetate saturated alcohol solution, then observed using transmission electron microscopy and images taken.

### 4.5. Detection of Skin Apoptosis by Terminal Deoxynucleotidyl Transferase (TdT) dUTP Nick-End Labeling (TUNEL) Staining

Skin paraffin sections were produced in the same way shown in the pathological observation. TUNEL staining was performed (Boster, Wuhan, China) according to the manufacturer’s instructions. Briefly, skin paraffin sections were dewaxed in xylene, rehydrated in graded ethanol, then treated with proteinase K solution in a wet box, and washed with 0.01M TBS. Then sections were treated with a TUNEL reaction mixture in a wet box and washed with 0.01M TBS. Finally, the sections were treated with Enhanced HRP-DAB Chromogenic Substrate Kit (Tiangen, Beijing, China), stained with hematoxylin, rehydrated in graded ethanol and xylene, and mounted. The primary antibody was replaced with PBS as a negative control experiment, and the remaining experimental steps were the same as above. The skin tissue sections were observed and photographed using the slide scanner Olympus VS120 (Olympus, Japan).

### 4.6. Transcription of Genes Related to Skin Apoptosis and Keratinization

The total RNA was extracted according to the manufacturer’s instructions in an RNAsimple Total RNA Kit (Tiangen, China). The concentration of the total RNA was determined using NanoDrop One^C^ (ThermoFisher Scientific, Shanghai, China). The total RNA was reverse transcribed into cDNA using a reverse transcription kit (Bao Biological Engineering Co., Ltd., Dalian, China). The sequences of the target genes were obtained from the NCBI database (https://www.ncbi.nlm.nih.gov/, accessed on 1 April 2022). Primers were designed (Table 2) and tested to determine the optimal annealing temperature and specificity. qRT-PCR reactions were performed using a Roche Light Cycler 480 (Roche, Switzerland). qRT-PCR conditions were as follows: 95 °C for 3 min, followed by 40 cycles of 94 °C for 5 s, and annealing at the optimal temperature for each primer pair for 30 s, then melting curve analysis. β-actin was selected as the reference gene [66]. The relative level of gene expression was calculated using the 2^−∆∆Ct^ method [67].

### 4.7. Immunohistochemistry

Skin paraffin sections were produced in the same way shown in the pathological observation. Immunohistochemistry was performed as previously described [28]. Briefly, skin paraffin sections were dewaxed in xylene and rehydrated in graded ethanol. After being washed with PBS, the sections were covered by 3% H_2_O_2_ for 10 min, blocked with 5% goat serum at room temperature for 20 min, and incubated with 50 μL rabbit anti-Bcl-2 antibody (Boster, Wuhan, China, 1:400 dilution) for 20 h at 4 °C, followed by 50 μL HRP conjugated goat anti-rabbit (Boster, Wuhan, China, 1:1500 dilution) for 1 h at 37 °C. Finally, the sections were treated with Enhanced HRP-DAB Chromogenic Substrate Kit (Tiangen, Beijing, China), stained with hematoxylin, rehydrated in graded ethanol and xylene, and mounted. The primary antibody was replaced with PBS as a negative control experiment, and the remaining experimental steps were the same as above. The skin tissue sections were observed and photographed using the slide scanner Olympus VS120 (Olympus, Japan). IOD determined the expression level of Bcl-2 protein in each group. For each section, five fields (scale bar = 20 μm) from each area of the image were analyzed using Image-Pro Plus 5.1 software (Media Cybernetics, Bethesda, MD, USA) [63].

### 4.8. Data Analysis

The data were presented as the mean ± standard deviation (M ± SD). Statistical significance was determined by a one-way analysis of variance (ANOVA) using GraphPad Prism version 9.1.2 software (GraphPad Software, San Diego, CA, USA). A probability (*p*) less than 0.05 or 0.01 was considered significant (* *p* < 0.05 or ** *p* < 0.01). The graphs were produced using GraphPad Prism version 9.1.2 software (GraphPad Software, San Diego, CA, USA).

## 5. Conclusions

After the rabbits were artificially infested with *S. scabiei*, obvious crusts appeared at 1 wpi and obvious clinical signs appeared at 4 wpi. These signs were similar to those of crusted scabies in humans, suggesting that rabbits may be an ideal animal model for studying crusted scabies. Following infestation, the mitochondria of skin epidermal cells were swollen, there was observed thickening of the epidermal layer, and there was an increase in the level of skin apoptosis. The process of skin crust formation was related to apoptosis and keratinization. These results may help us to further understand the mechanisms by which the host skin crust is formed in response to *S. scabies* invasion.

## Figures and Tables

**Table 1:** Pathological damage scoring criteria for rabbit skin.

Score	Scab	Epidermal Thickening	Visible Mites	Inflammatory Cell Infiltration	Congestion and Hemorrhage
0	not observed	not observed	0	not observed	not observed
1	slightly	slightly	1–2	slightly	slightly
2	moderately	moderately	3–5	moderately	moderately
3	severely	severely	≥6	severely	severely

**Table 2:** Primer sequences used by qRT-PCR.

Genes	Accession no.	Primers	Sequence (5′–3′)	Tm (°C)
β-actin	NM001101683.1	Forward	GGCATGGAGTCGTGTGGCATC	62
		Reverse	CGTGTTGGCGTACAGGTCCTTG	
Caspase-1	XM_008262043.2	Forward	ATGCCTGGTCTTGTGATGTGG	57
		Reverse	GTACAGGATGATAGCACTCTTGGC	
Caspase-3	NM_001082117.1	Forward	GCAAATCAATGGACTCTGGGAAA	56
		Reverse	CGGGACGACATTCCAGTGTT	
Caspase-8	XM_017343029.1	Forward	GGTTGCAGCTACGTTCTCCT	56
		Reverse	GATGGGCTCCTGCTTCCTTT	
Caspase-10	NM_001099966.1	Forward	CCAAAGAGGAAGTGGAGCGT	57
		Reverse	GTTCATCTCGGTTCTGGGCA	
Caspase-14	XM_017338752.1	Forward	CTGAGGTGAGCCCAGACAAA	57
		Reverse	GTTTTCGGAGGGTGCTTTGG	
Bcl-2	XM_008261439.2	Forward	ACCAGGAGGCAAAGAGCATC	57
		Reverse	CAGAGATGGTGGGGTTTCGT	
keratin 1	XM_002711004.3	Forward	TTCGTGTCGGCCACTTACTC	57.5
		Reverse	CACCTTCCTCCCTGCAATCA	
keratin 5	XM_002711001.3	Forward	CGAGCTCCGTTCTGTTCTCT	58
		Reverse	AGACACTAGACTGGCGAGACA	

## Data Availability

The datasets used or analyzed in the current study are available from the corresponding author upon reasonable request.
